# Malignant Tumor of the Inferior Maxilla

**Published:** 1845-05

**Authors:** 


BIRMINGHAM PATHOLOGICAL SOCIETY,—JAN. 4TH, 1845.

*Malignant Tumor of the Inferior Maxilla.*—Mr. Hodgson exhibited a portion of the lower jaw, which he had removed by operation, affected with malignant disease, which had commenced in the laminated structure of the bone, and not, as is usual, in the medullary membrane. Mr. Freer, House-Surgeon to the General Hospital, supplied the following notes of the case:—

A. Ward, aged 50, of mixed temperament, and has always enjoyed tolerable health. In the commencement of October she first perceived a small excrescence between the canine and cuspidate teeth on the left side of the inferior maxilla, and she suffered occasional lancinating pain, with bleeding; it rapidly increased in size, pressing the teeth forwards, and spread over the double teeth on the same side. Early in November she had three teeth extracted, the tumour speedily filling up the space. On the 14th of November the tumour extended from the level of the angle of the inferior maxilla to the symphysis: its level was considerably above that of the surrounding teeth, and it projected considerably into the mouth. The bone was not thickened, nor were the integuments or neighbouring glands at all implicated in the disease.

The tumour was cleanly scooped out so as to leave the alveolar structure denuded; its texture was the same as the specimen produced. The diseased structure did not appear to again encroach until the 1st of December, when it was necessary to daily destroy the new growth with escharotics, and these became insufficient to destroy it by the middle of December. The tumour continuing to encroach, Mr. Hodgson removed the portion of the left inferior maxilla from the symphysis to the angle.

Mr. Hodgson said that it appeared to be a disease of the medullary membrane. It was quite scooped out, by which every particle of disease seemed to be removed. It soon, however, showed itself again, when it was destroyed by nitric acid. After this it very soon came on again, and at the end of the week it had very much increased, which gave the idea that the disease originated in the medullary membrane. It was thought advisable to remove the diseased portion of the bone, and consequently the operation was performed this morning. Mr. Hodgson then described the operation. With respect to the dangers of the operation, Mr. Hodgson said that it had been stated that there was much danger of the patient being suffocated from the retraction of the tongue, and its closing the glottis at the time of division of the muscles connecting it with the maxilla. He had operated three times, and had not been aware of any such occurrence, and he did not think it had been noticed by other operators. He should rather attribute any symptoms of suffocation to the blood having found its way into the larynx, a circumstance not at all uncommon in operations connected with the tongue and palate.

